# Green Extraction Processes for Complex Samples from Vegetable Matrices Coupled with On-Line Detection System: A Critical Review

**DOI:** 10.3390/molecules27196272

**Published:** 2022-09-23

**Authors:** Francisco W. Maciel-Silva, Daniel Lachos-Perez, Luz Selene Buller, William G. Sganzerla, Montserrat Pérez, Mauricio A. Rostagno, Tania Forster-Carneiro

**Affiliations:** 1 School of Food Engineering (FEA), University of Campinas (UNICAMP), Campinas 13083-862, SP, Brazil; 2 Department of Chemical Engineering, Federal University of Santa Maria, Santa Maria 97105-900, RS, Brazil; 3 Department of Environmental Technologies, University of Cadiz, Campus de Puerto Real, S/N, Puerto Real, 11500 Cadiz, Spain; 4 School of Applied Sciences (FCA), University of Campinas (UNICAMP), Limeira 13484-350, SP, Brazil

**Keywords:** sample preparation, green extraction techniques, on-line coupling, fully automated analysis, bibliometric analysis

## Abstract

The detection of analytes in complex organic matrices requires a series of analytical steps to obtain a reliable analysis. Sample preparation can be the most time-consuming, prolonged, and error-prone step, reducing the reliability of the investigation. This review aims to discuss the advantages and limitations of extracting bioactive compounds, sample preparation techniques, automation, and coupling with on-line detection. This review also evaluates all publications on this topic through a longitudinal bibliometric analysis, applying statistical and mathematical methods to analyze the trends, perspectives, and hot topics of this research area. Furthermore, state-of-the-art green extraction techniques for complex samples from vegetable matrices coupled with analysis systems are presented. Among the extraction techniques for liquid samples, solid-phase extraction was the most common for combined systems in the scientific literature. In contrast, for on-line extraction systems applied for solid samples, supercritical fluid extraction, ultrasound-assisted extraction, microwave-assisted extraction, and pressurized liquid extraction were the most frequent green extraction techniques.

## 1. Introduction

The extraction of bioactive compounds from natural substrates for food, pharmaceutical, and cosmetic applications can be advantageous for producing high value-added products and for nutrients recycling from industrial byproducts and waste [1,2,3], as a circular economy strategy [4,5,6]. Furthermore, the current extraction techniques coupled with on-line detection can be considered more environmentally friendly than conventional techniques [7,8,9].

Currently, most extraction techniques are operated off-line to the analysis system [10,11]. Nevertheless, these processes could be integrated into an on-line system for several cases, where the entire analytical procedure takes place in a closed complex—usually automated. On-line systems could notably provide manifold benefits, including shorter analysis time, better reproducibility, improved detection limits, sensitivity, and results reliability. In addition to avoiding some problems associated with the traditional approach, and its consequent experimental error propagation, on-line sample preparation systems tend to be more productive and less prone to accuracy and precision errors [12].

The sample preparation step aims to isolate the target analytes and to improve the selectivity, precision, and repeatability of the analysis. This step generally includes extraction, concentration, and cleaning procedures for impure and complex samples [13,14]. Meanwhile, these procedures could represent a significant disadvantage, since individual preparation steps substantially increase the risk of sample loss and possible sample contamination, leading to reduced analysis reliability [7,14].

When considering sample preparation and chromatographic analysis, the first is the most time-consuming, tedious, and error-prone step [13], commonly considered by researchers as the bottleneck of the whole analytical procedure [10]. The analysis automation potentially improves the measurement precision, sensitivity, and accuracy, also reducing the consumption of organic solvents [7].

Accordingly, several techniques based on dynamic systems were developed to meet the imminent demand for fastness, sensitivity, repeatability, and reliability [15]. The main available green technologies with coupling capability for on-line detection systems are solid-phase extraction (SPE), supercritical fluid extraction (SFE), liquid-liquid extraction (LLE), microwave-assisted extraction (MAE), ultrasound-assisted extraction (UAE), pressurized liquid extraction (PLE), accelerated solvent extraction (ASE), supercritical water (SCW), and subcritical water (SubCW) [9,10,14,16,17,18,19].

The above-mentioned technologies are more efficient than conventional technologies (maceration, decoction, and Soxhlet extraction) with respect to many aspects, such as lower time demand, higher selectivity, and lower solvents and reagents consumption [12]. These modern technologies are commonly coupled with liquid chromatography (LC), gas chromatography (GC), supercritical fluid chromatography (SFC), and capillary electrophoresis (CE) [11,20]. The SPE and LLE are best suited for liquid samples, as long as the others are usually applied to solid samples [7,15].

Automated flow-based sample treatment systems and separation techniques appear to be a trend in analytical chemistry coupling systems, provided that remarkable assertiveness of analysis results could be achieved [10,11,12]. Moreover, coupled systems are likely to increase the analytical throughputs and efficiency in diversified and easier ways to use on-line systems [21] in a wide range of applications; for instance, for lipophilic samples using SFE with separation based on carbon dioxide (CO_2_) [9,22], and food drinks with juices, beer, coffee, tea, and water, mainly using the SPE technique [11].

In this context, this article presents a systematic review of extraction systems coupled with on-line analysis systems for solid matrices from vegetable origin. The prevailing publications on extraction processes associated with on-line analysis techniques from 1990 to 2021 were collected and examined. In addition, to evaluate the documents on this topic, statistical and mathematical methods were applied to assess the timeline evolution and future trends of this research field through a longitudinal bibliometric analysis. The results of this review allow the identification of the current state-of-the-art coupling systems for green bioactive compound extraction.

**Figure 1:**
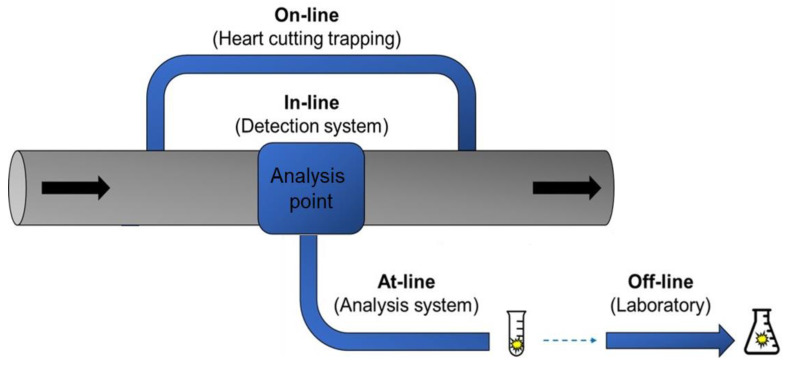
Classification of coupling forms with the detection system.

The extraction technologies can be coupled with detection techniques in various ways, depending on the degree of software/hardware automation and human intervention [23]. Accordingly, Figure 1 illustrates the coupling forms, classifying them as off-line, at-line, in-line, and on-line.

The off-line coupling approach includes manual injection of the pre-processed sample into the analysis equipment [24]. This procedure is appropriate for a small number of samples, when conventional methods are expected to be sufficient and there is no need for automated methods [13]. The advantage of carrying out separated processes is a kind of increased flexibility. For LC analysis, it is possible to minimize interferences and undesirable effects related to dirt samples, which may cause bandwidth expansion, overlapping peaks, co-elution with empty volume, and peak asymmetry. Nevertheless, this procedure presents the lowest degree of automation, requiring more time and labor, as well as a risk of sample loss or contamination, which would deliver a lower analysis sensitivity and precision [12].

For at-line coupling, the analyzer is disconnected, yet close to the process [25]. Generally, at-line coupling procedures include a programmable robotic station to link the flow system to the separation or detection instrument. Usually, the pre-treated sample is delivered from the flow system to a vial or to the injector of the analytical instrument [24]. It enables automation and allows for performing a parallel analysis to the flow system with a high precision level, little labor requirement, and low analysis time. Nevertheless, the risk of sample loss or contamination is considered high for this approach [13].

In-line coupling involves complete and close integration of the detection or separation instrument into the flow system [23]. The analyzer is equipped with probes inserted into the process stream, and the analysis is performed in situ [25]. The in-line procedure is entirely automated, promoting more injections while the process is still in progress, which allows a faster response in the feedback cycle. It is possible to implement an automatic periodic cleaning (“self-cleaning”) routine. However, a significant disadvantage is its inability to manipulate samples in parallel because the overall analytical process is restricted to the integrated flow device. Therefore, it does not apply to many methods [12].

The on-line coupling strategy implies direct contact between the flow system and the components of the detection equipment, usually through a flow bypass [11,21]. The analyte from the sample processed in the flow system is transported to an interface and introduced into the pressure-driven separation system in CE [26], mobile phase in LC [14,20] and carrier gas in GC [27]. This configuration enables sequential analysis and high automation capacity, requiring little labor. It is subject to a low possibility of sample loss or contamination, promoting high precision and sensitivity in the analyses. In turn, it requires sophisticated equipment and software, representing high installation costs [12,13]. Similar to in-line methods, a “self-cleaning” routine is possible for on-line methods.

Among other requirements, the robustness of an analysis system under specific process conditions represents an important issue. The demand for in-process analysis equipment is high; the limits of accuracy and detection are often lower than state-of-the-art laboratory equipment. However, the analyses used are typically adapted to the process-specific requirements, leading to highly specialized equipment incorporated into sophisticated control circuits that would allow quality control in real time [23].

Moreover, various technologies are capable of promoting the extraction of bioactive compounds, obtaining a large variety of biochemical products, in addition to platform chemicals. Most of them are viable for foods, pharmaceuticals, and cosmetics [28]. Frequently using these technologies is a relevant strategy for developing a circular economy, since its exceptional advantage in valorizing agro-industrial waste is notable [29]. Furthermore, many current extraction technologies enable on-line or in-line detection system coupling, which is considered more environmentally friendly than conventional technologies [14]. A common trend in extraction technology is to replace the use of toxic reagents with reagents obtained from renewable sources, as well as the integration of analytical processes and operations, saving energy, and reducing the use of reagents [30].

## 2. Materials and Methods

### 2.1. Data Collection

The bibliometric analysis retrieved publications from the Web of Science (WoS) core collection, from Clarivate Analytics, using the Science Citation Index Expanded (SCI-Expanded) option. The WoS database presents extensive and multidisciplinary coverage of bibliographic data from leading scientific publications. Consequently, it is a primary information source for academic and bibliometric studies [31,32].

The keywords used to identify the articles in the area of interest were based on a scientific literature search. The terms selected to collect information related to the extraction processes were “*solid-phase extraction*”, “*liquid-liquid extraction*”, “*supercritical fluid extraction*”, “*pressurized liquid extraction*”, “*supercritical water*”, “*subcritical water*”, “*accelerated solvent extraction*”, “*superheated water extraction*”, “*microwave-assisted extraction*” and “*ultrasound-assisted extraction*”. The terms used for the detection techniques were “*chromatography*”, “*HPLC*”, “*LC*”, “*GC*”, “*UV-detection*”, and “*supercritical fluid chromatography*”. Finally, to assess the integration of processes and detection techniques into an on-line system, the keywords selected were “*on-line*” and “*in-line*”.

The bibliometric research was carried out on January 13, 2022, using the TOPIC search field, which combines words in titles, abstracts, author keywords, and keywords defined by the WoS database (keywords plus). The strategy of placing the terms in quotation marks was used to limit the search to publications that contained those terms in that exact word order. The Boolean operators OR (union of terms in groups) and AND (providing an intersection between groups) were used to select the final dataset. A total of 2633 documents published from 1990 to 2021 were found. Furthermore, a refining step was performed to choose only articles and reviews, for which 2353 files were obtained (2146 articles and 207 reviews). The Supplementary Material illustrates the methodology synthesis applied for the data collection and bibliometric analysis.

### 2.2. Data Processing

The bibliometric analysis was performed using the “Analyzing Results” tool provided by WoS. The number of publications, year of publication, number of citations, types of documents, research areas, and countries were gathered. The graphic illustrations were generated using Origin 8.0 software from OriginLab^®^. The construction of bibliometric maps based on co-authorship was created using the VOSviewer© 1.6.13 software, providing the graphical representation of bibliometric maps to facilitate data interpretation [33].

## 3. Results and Discussion

The bibliometric analysis broadly presented all works related to “*on-line*” and “*in-line*” coupling systems for: *(i)* extraction techniques with analysis systems with or without sample purification/cleaning techniques; *(ii)* extraction techniques with sample purification/cleaning techniques, and *(iii)* sequential analyses. A detailed bibliometric analysis results description and discussion follows in the next sections.

### 3.1. Scientific Development of On-Line and In-Line Coupling

**Figure 2:**
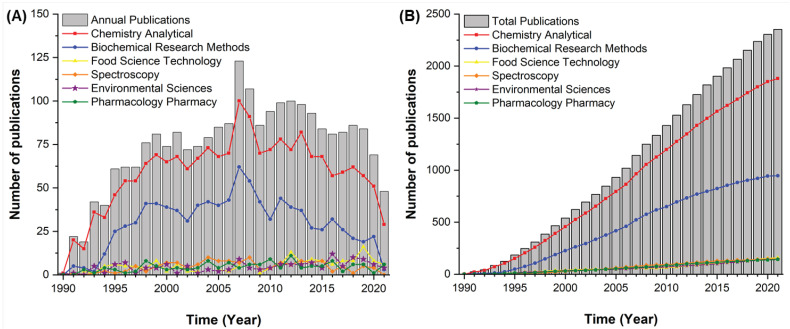
Evolution of the number of publications and main research areas. (**A**) Annual publications and (**B**) total publications.

The scientific literature data collection regarding on-line and in-line coupling is proven to be relevant worldwide, covering 67 different countries in the total publication period assessed. Figure 2 shows the evolution of the number of publications and highlights the main research areas.

Figure 2A emphasizes the advancements in annual publications, while Figure 2B focuses on the total breakthrough of the works performed. The publishing started in 1990 with only one document, and rapid growth in the following years is observed. The publishing peak occurred in 2007, with 123 studies. Since 1995, studies in this area reached the mark of more than 60 annual publications, exposing the broad scope and the particular importance of this area for the international scientific community. From 2005, more than 80 yearly publications were identified, except for the years 2020 and 2021, in which only 69 and 48 papers were found, respectively (a decrease may be associated with the COVID-19 pandemic).

In the 32 years of publications in this field, a total of 2353 papers were produced, arranged in six main research areas according to the Web of Science categories: *“Chemistry Analytical”*, *“Biochemical Research Methods”*, *“Food Science Technology”, “Spectroscopy”, “Environmental Sciences”,* and *“Pharmacology Pharmacy”*. These categories played an essential role in the development of this subject. Since the beginning of publications, the *Chemistry Analytical* area led the ranking, followed by *Biochemical Research Methods*. The other areas of the top six have a similar number of papers, and they change positions in the ranking over the years. The *Food Science Technology* area currently occupies the third position, with 158 articles. Fourth, it is the *Spectroscopy* area with 146. *Environmental Sciences* is in fifth with 145, and in sixth is *Pharmacology Pharmacy* with 143. The *Environmental Sciences* area is currently in evidence due to increased environmental concerns.

### 3.2. International Collaboration

**Figure 3:**
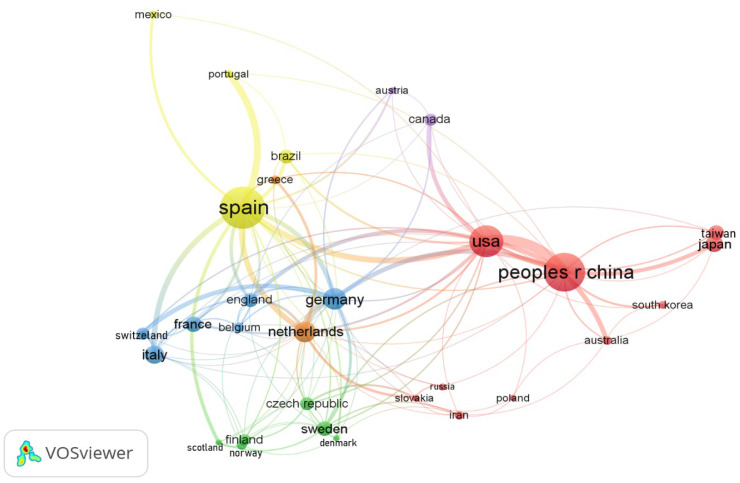
Collaboration map among the top 30 most productive countries.

Research partnerships between the leading countries in the on-line and in-line coupling areas were evaluated. The evaluation of international collaboration was carried out through the bibliometric analysis applied to co-authorship and the number of cumulative publications with chosen thresholds to understand how the connections were established. Figure 3 shows a bibliometric network in which the size of the marker is proportional to the number of accumulated publications, as well as the thickness of the lines that make the connections between the stamps, and the colors indicate to which cluster the item belongs. Furthermore, it is noteworthy that the number of subjects was limited to display only the top 30 countries.

The bibliometric map presented in Figure 3 shows six clusters among the thirty most productive countries. The red-colored cluster has the largest number of countries (corresponding to 10 items). The clusters with the least number of items are the orange and purple ones, both with only two countries. The group responsible for the smallest number of publications is purple, accounting for 72 articles. The cluster with the most considerable incidence (red) accounts for 970 articles, and the second-largest, the yellow one, presents 560 reports. Although there are two clusters with six countries and the yellow one contains only four, the last is placed in the second position in the number of publications containing Spain, which is the most productive country.

Spain, China, and the USA significantly contributed to this research field, with 19.29%, 17.30%, and 11.81% of the scientific recordings, respectively. These countries published a considerable number of scientific articles collaboratively with other countries. However, when analyzing the number of international collaborations, the rank varied as follows: the USA started as the leader, having published in partnership with 32 countries, followed by Spain, with 31 countries; the third country is Germany, which collaborated with 24 countries. Finally, China dropped to the fourth position, with 23 international collaborations.

### 3.3. Journals with More Publications and Most Cited Articles

All of the articles found in the research area of extraction with on-line detection systems were published in 282 different journals, which illustrates the diversity of publications and broad interest in this research field from multiple perspectives. Notwithstanding, most journals (approximately 91%) published fewer than 20 articles, accounting for only 25 journals publishing 20 or more reports. Otherwise, this set is responsible for approximately 71% of the publications. Consequently, it can be inferred that those 25 journals are the most chosen for this research area. According to Bradford’s law, this distorted distribution is expected in the bibliometric analysis, since journals are dedicated to specific topics [34] and about 500 to 1000 journals are needed to cover 95% of the “significant” literature published in a given field [35]. Accordingly, Table 1 presents the ten most active journals responsible for 53% of the publications.

From Table 1, it can be observed that the *Journal of Chromatography A* leads the ranking with approximately 16% of the total publications, more than double the publications in *Talanta*, the second journal in the ranking. Both journals belong to the company *Elsevier*, which also corresponds to the third, fourth, and eighth journals in the ranking. This publisher is responsible for the four journals with the most significant number of publications and accounted for five journals in the top ten. Among the top ten journals, the other publishing company is ACS Publications, responsible for the fifth ranking position, with the most significant impact factor (6.986). Springer, responsible for two journals, tied for the sixth position in the ranking. Finally, the Wiley Online Library is responsible for the ninth and tenth positions.

The analysis of the ten most cited articles until 2021 (Table 2) revealed no direct relationship between citations in a specific publication and the most active journals in the area. The most cited article was published by *Chemosphere*, which is not among the top ten journals, occupying the 23rd position in the ranking and accounting for 18 publications. On the other hand, the *Journal of Chromatography A*, the journal’s ranking leader (Table 1), was the only journal with more than one publication among the top ten most cited articles.

The work presenting the highest number of citations is a critical review focused on phosphorus flame retardants [37], which dealt with properties, production, environmental occurrence, toxicity, and analysis issues. This large variety of issues may explain why different researchers from different areas cited it.

Finally, five of the most cited articles [37,39,40,44] do not belong to the top ten journals in terms of publications. In addition to the first in the ranking and the third most cited article [39], which was published by *Trends in Analytical Chemistry*, the journal presents 34 publications in the on-line coupling field and ranks thirteenth. The other articles are the fourth [40] and the eighth [44] on the list, belonging to the *Accounts of Chemical Research* and *Glycoconjugate Journal*, respectively. Both journals have only one article in this field of study. Finally, the 10th position [46] belongs to *Environmental Science & Technology*. This journal presents 20 articles in the field and occupies the 25th position in the ranking of the top journals.

Beyond the fifth, sixth, and seventh, the first three most cited articles belong to Elsevier journals, totalizing six reports from the list. The fourth, ninth, and tenth articles belong to ACS Publications, and the eighth article is from *Springer*. The top five publishers in numbers of published articles are Elsevier, Wiley, Springer, ACS Publications, and the Royal Society of Chemistry, with 1204, 290, 183, 141, and 102 articles, respectively. Another critical detail in Table 2 is that most of the included works are reviews, and only three documents refer to research articles (eighth, ninth, and tenth).

### 3.4. Keyword Analysis

The keywords are essential sources of information because they provide evidence for the issues and central themes in a publication [47]. The terms used as keywords can be used to investigate the research trajectory, to analyze trends in a research area, and to investigate the existing knowledge gaps in a certain scientific field [31]. Therefore, the 30 main authors’ keywords and keywords plus (provided by WoS, based on a unique special algorithm to increase the power of cited reference search) of the research field analyzed in this review are highlighted in Table 3.

The examination of the keywords in the 32 years of publication revealed that 8626 terms were already used to characterize searches about on-line and in-line coupling (with 4679 author keywords and 4927 keywords plus), suggesting the existence of a great diversity of research themes. Another argument reinforces this hypothesis: the VOSviewer software frequency analysis revealed that 6042 keywords were used only once. According to Chuang, Huang, and Ho [48], many unique keywords probably indicate a lack of continuity and a significant disparity in the research focus.

Table 3 shows that the term most used was “*Solid-phase extraction*”, either when chosen by the authors or the WoS. In addition to this term, the two columns present eighteen (18) repeated keywords. It is also possible to observe that the keywords plus have more occurrences than the authors’ keywords. Furthermore, 77.4% of the terms in the authors’ keywords appeared in only one article, and that number was 64.3% for keywords plus.

It is possible to consider three main groups of keywords in the study area: samples, process techniques, and analysis techniques.

The main terms related to the sample group are *“Urine”*, *“Plasma”*, *“Human plasma”*, *“Pesticides”*, *“drugs”*, *“residues”*, *“Waste-water”*, *“Water”*, and *“Water samples”*, suggesting that automation using on-line and in-line coupling is more advanced in the area of health, water treatment, and food products.

For process techniques, there are general terms, such as “Sample preparation”, “Preconcentration”, “On-line pre-concentration”, “Extraction”, “On-line extraction”, “Separation”, and “Microextraction”. In addition, specific terms, such as “Solid-phase extraction”, “On-line solid-phase extraction”, “On-line SPE”, and “Supercritical fluid extraction” are present. Currently, SPE is one of the most used techniques for the extraction and/or concentration of complex samples, allowing analytes in deficient concentrations to be detected by methods such as high-performance LC, GC, and CE [11,20].

In general, the use of the word “*on-line*” is more performed by the authors. Moreover, for the keywords plus, the term appeared disassociated with the process technique. For plus keywords, the analysis of the association between them, using VOSviewer software, showed that the term *“Solid-phase extraction”* appeared highly associated with *“High-performance liquid chromatography”*. For the author keywords, the term “*Solid-phase extraction”* appeared more associated with *“Capillary electrophoresis”*.

The main terms of the analysis techniques group are “Mass spectrometry”, “Capillary electrophoresis”, “Liquid chromatography”, “Tandem mass spectrometry”, “Chromatography”, “Identification”, and “Quantification”. It was found that among the keywords of this group, there is an extensive use of synonyms, such as “Liquid chromatography”, “High-performance liquid chromatography”, “Column liquid chromatography”, “HPLC”, “LC-MS”, and “LC-MS/MS”. The two last terms mean LC coupled to mass spectrometry, with one or two detectors, also known as tandem mass spectrometry.

## 4. Innovative Extraction Techniques Suitable for On-Line Coupling

From the bibliometric analysis results, it was possible to collect, to review, and to assess state-of-the-art on-line coupling systems. The main findings are further scrutinized and critically analyzed.

Several extraction systems can be coupled on-line with analysis systems using liquid and solid samples. Liquid samples are more straightforward to operate than solid samples, considering that on-line sample preparation techniques are broadly based on continuous flow approaches (which are especially suitable for liquids).

**Figure 4:**
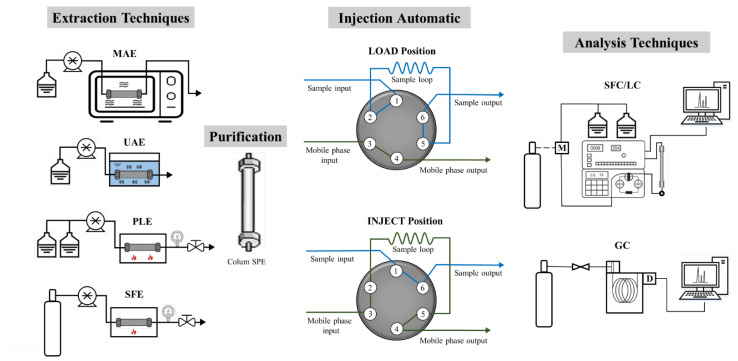
Simplified diagram of on-line coupling among the main extraction and analysis techniques.

Among the extraction techniques for liquid samples, SPE is the most common in combined systems [7,13]. In SPE, the compounds dissolved or suspended in a liquid mixture are extracted using an interface coupled or not with solid extraction techniques and chromatography [49]. Otherwise, the solid matrix analytes are first extracted through polar or nonpolar solvents before the analysis. The most common on-line extraction systems for solid samples are SFE, UAE, MAE, and PLE. The coupling is usually performed with the aid of multiport valves. In cases where the extracts have a high content of soluble solids or a high concentration of impurities, SPE is used for in-line purification [50]. Figure 4 shows a simplified diagram of the primary extraction and analysis techniques for on-line coupling.

On-line extraction systems can be carried out in either static or dynamic mode. In the context of transferring the extract to the analytical system, the dynamic mode is the most common, and the analytes are removed continuously from the sample to the fluid (solvent). Furthermore, as the sample is continually exposed to the solvent, the transfer of analytes from the sample matrix to the solvent is enhanced [13]. Therefore, for solid samples, low flow rates and extract volumes of less than 1–2 mL must be kept. Otherwise, the extract must be concentrated before transferring it to the analytical system.

Another option is in-line SPE coupling. Several works in the literature demonstrated that SPE considerably improves concentration and sensitivity when diluted samples are analyzed and makes it possible to analyze complex matrices by extracting solids, enabling simple coupling [51]. The solvent (or fluid) is continuously washed through the SPE extractor at a constant flow rate for a specified period.

Furthermore, the solvent (or fluid) must be compatible if the analytical system is chromatographic (mobile phase for LC and carrier gas for GC). It is essential to highlight that the solvent (or fluid) must be sufficiently volatile and preferably nonpolar when coupled with the GC. On the other hand, the extracts should preferably be a weak strength eluent for coupling with the LC system [7,13]. The following sections will delve into extraction systems coupled on-line with analytical techniques for vegetable origin solid matrices.

### 4.1. Supercritical Fluid Extraction

SFE is an attractive, green, novel technique that uses supercritical fluids (typically carbon dioxide). Due to its selectivity in adjusting the temperature or pressure, it is possible to obtain extracts with a low concentration of undesired compounds. Moreover, with the addition of a modifier to CO_2_, termed cosolvent, the efficiency of the extraction of more polar analytes is possible and a wide variety of compounds of different polarity can be recovered. In this regard, it is essential to highlight that SFE is hardly matrix-dependent, and therefore, the chosen chromatographic technique will depend on the sample and analytes chemical composition [52,53].

SFE is well-suited for on-line coupling systems (LC, GC, and SFC), considering that CO_2_ becomes gaseous upon depressurization and it is easily removed from a flow system. Additionally, SFE produces clean extracts with minimal residual organic solvents and usually does not require additional analytes pre-concentration before the chromatographic analysis [7,52]. In general, on-line coupling systems employ intermediate trapping, multiport valves, and one or more pumps for the dynamic extraction or for transferring the extract to the chromatographic system. As mentioned above, on-line coupling systems can be carried out in either static, dynamic, or as a combination of both modes. Table 4 presents the different SFE on-line coupling systems, conditions (feed, pressure, and temperature), static and dynamic modes of different samples, and detectors.

On-line SFE coupled to LC is technically a challenge due to the incompatibility of the technique that produces a large volume of gas (normally CO_2_) while using liquid as a mobile phase. Therefore, a crucial requirement is to keep a gas-free mobile phase to avoid bubbles that promote an unstable pump performance and detector sensitivity loss [57,61,62,63]. Hamada et al. [55], studied the coupling system SFE-SPE-LC-MS for on-line extraction and trap columns in green, yellow, and red peppers, presenting excellent results with the concentration column with a six-port valve. The interfaces based on a split/splitless injector, an on-column injector, or a programmable temperature vaporizer were less common in on-line SFE coupled to LC than in SFE coupled to SFC [27,64,65,66,67].

Zhang et al. [16] presented a coupling system SFE-SFC with a photodiode array detector (PDA) for the rapid determination of oleanolic acid and ursolic acid in *Chaenomelis Fructus* within 40 min (10.79 mg/g dry plant, Rs = 2.36). Additionally, coupling the SFE-SFC system with the PDA detector, compared with other off-line methods obtained, higher extraction yields for all four aromatic constituents of vanilla beans. The direct on-line extraction and determination by SFE with chromatography and mass spectrometry were studied for apocarotenoids and carotenoids compound extraction. The results show a very fast extraction yield at 80 °C, in less than 17 min for monoesters and diesters carotenoids [59]; and apocarotenoids and apocarotenoids fatty acid esters were detected in yellow tamarillo through conventional and sub-2-micron particle C30 columns [56].

Although the on-line SFE-GC system might seem simple, the flow rate of the CO_2_ increases after depressurization, and the volume of the gas is significant, the only coupling is found at the output of the system, not through an interface valve. Handling such a high flow rate and a large gas volume requires careful selection of the interfacing technique, if pressurized. Finally, SFC is an analytical technique that uses supercritical fluid as a mobile phase. Although SFC has some advantages, such as high resolution and high throughput, its development and application are not widely used as GC and LC, due to some aspects related to instrumental particularities and a complex handling of diverse operational variables, which can lead to a complicated optimization process [58,68,69,70,71].

### 4.2. Ultrasound-Assisted Extraction

Ultrasound is an intensification extraction technique widely used to improve the removal of analytes from a solid sample matrix. UAE is widely applied due to faster extractions, high reproducibility, simplification of manipulation and processing, and higher purity of the final product, allowing lower consumption of solvents and energy compared to conventional extraction techniques. Only five works approaching UAE systems coupled on-line with GC were identified [72,73,74,75,76]. Table 5 presents UAE coupled systems’ operational conditions (retention time, pressure, and temperature) for different samples.

Zhang et al. [77] studied a novel UAE with an on-line solvent concentration system highly automated. The results show the extraction of several chemicals by coupling parallel counter-current chromatography. This UAE system has unique advantages, where extraction of materials and purification of the extract can be simultaneously achieved, thereby reducing the “liquid-liquid” purification steps. The process also enables continuous feeding, slag discharge, and liquid discharge, which improves the extraction [81]. Falkova et al. [78] studied an automated flow batch-based determination of anthraquinones with on-line ultrasound-assisted surfactant-mediated extraction by one sequential injection and another stepwise injection. The results show sampling frequencies of 12 h^−1^ for SIA and 6 h^−1^ for stepwise injection, respectively. Similar studies with continuous UAE incorporated into an on-line flow injection manifold were used for the determination of zinc in meat samples by flame atomic absorption spectrometry, and the flow injection methodology allowed a sampling frequency of 80 samples per hour [82].

The cited works presented on-line combinations in dynamic mode: *(i)* a predetermined volume of solvent flows through the solid matrix that is static and fixed in the extractor, termed a semi-continuous process, and *(ii)* solvent and the solid matrix continuously flow through an ultrasonic bath, termed a continuous process.

Dynamic extraction (semi-continuous or continuous) is favorable in many aspects: the analytes are removed as soon as they are transferred from the solid matrix to the solvent, and the continuous exposure to fresh solvent enhances the transfer of analytes from the sample matrix to the solvent.

### 4.3. Microwave-Assisted Extraction

MAE is a well-established green extraction technique widely acknowledged as rapid, easy to operate, and efficient for solid and complex matrices [83,84]. The microwave energy heats the solvent in contact with the matrix, solubilizing the analytes. The solvent or even the sample must be dielectric. Therefore, the dielectric constant is one of the critical parameters associated with MAE. Due to this fact, it is crucial to select solvent mixtures to alter the dielectric constant until suitable characteristics are achieved for the extracted sample. Good extraction efficiencies are observed in the scientific literature. Several of them are similar to or higher than classical techniques, such as Soxhlet, SFE, or PLE [83]. Moreover, MAE solvent consumption and extraction time are lower than classical techniques, facilitating on-line analysis [84]. Table 6 presents MAE coupled techniques, operational conditions, methods (static mode and dynamic mode) for different samples, and composts of interest.

Gao et al. [85], studied on-line ionic liquid-based dynamic microwave-assisted extraction with high-performance liquid chromatography (DMAE-HPLC) to determine lipophilic constituents in an herbaceous substrate; the proposed method showed a homogeneous and stable analytes suspension when compared with the off-line ionic liquid-based DMAE and other methods. Additionally, another DMAE-HPLC coupling system showed optimal conditions for extracting Andrographis and Andrographolide for a flow of 1.0 mL/min, microwave power of 80 W, and extraction time of 6 min in 60% of aqueous methanol [87]. Wang et al. [86], studied DMAE coupled on-line with a cleaning up process (silica gel column) to remove chlorophyll from tea; the proposed method presented higher selectivity and sensitivity than conventional extraction methods, such as Soxhlet and LLE. In other studies, the analytes were automatically transferred from the SPE column to the analytical column, with a UV detector at 238 nm [51,87], a PAD detector [49] and a UV detector monitoring at 270 nm [86].

MAE may be a promising on-line coupling technique. Nevertheless, only limited research on food substrates were published [85,89].

### 4.4. Pressurized Liquid and Subcritical Water Extraction

PLE, also known as accelerated solvent extraction (ASE) or pressurized solvent extraction (PSE), extracts a sample using conventional solvents under high temperature and pressure. When the PLE technique operates with solvent water (or fluid) at different pressures, different terms can be used to define this technique: hot water extraction (HWE), subcritical water extraction (SubCWE), and pressurized hot water extraction (PHWE). PLE technique employs high temperatures and pressures that can increase the solvating power, decrease the viscosity, and increase the diffusion rate [2,90].

Therefore, PLE is a promising, environmentally friendly, and faster extraction technique than conventional solid–liquid extraction techniques, such as Soxhlet extraction. The temperature and pressure parameters improve the solubility, mass transfer, and increase the disruption of the surface equilibrium [2,91]. Table 7 presents the different PLE studies on vegetal origin solid samples with coupled on-line detection systems, detailing the sample used, analyte, PLE and LC conditions, and type of detection.

Some publications approaching SubCWE on-line with either LC or GC in a dynamic mode were reported, and the samples analyzed were soils, sediments, and wastewater [97,98,99,100,101]. The growing interest in the SubCWE might be associated with the fact that water is environmentally friendly and intimately related to LC. SubCWE systems coupled on-line with LC are frequently applied because the solubility of the analytes extracted in water decreases dramatically when subcritical water is cooled down; trapping analytes before LC analysis is therefore relatively accessible. The analytes are collected either to a solid-phase trap (SP-trap) for LC or GC, or even in a membrane extraction unit for GC. SubCWE-LC coupling with an SP-trap is simpler than SubCWE-GC coupling, since the extract from the SP-trap can be directly eluted to the LC column by an appropriate eluent [100,101]. Therefore, SubCWE successfully shows the potential of this integrated approach. SubCWE is inherently limited to using water, and its properties change in a subcritical state, where water above 300 °C behaves similarly to organic solvents [28].

The choice of analytical system type is primarily determined by the specific requirements of the sample, i.e., sample composition and complexity, and amount/concentration of analytes. For these reasons, the works with PLE presented on-line coupling only with LC, unlike SubCWE, which had studies using coupling with GC systems. This aspect is probably associated with the high eluent strength of the organic solvents usually applied for PLE. Then, the volumes used are usually significant to efficiently trap the analytes into the SP-trap sorbent [13,93]. Therefore, methods that can effectively couple PLE to chromatography via on-line solid-phase trapping/pre-concentration would be beneficial to explore. Some works were reported using on-line coupling PLE-LC systems [102,103], and those that used solid samples of plant origin are detailed in Table 7.

## 5. Future Directions

From this systematic review, it is clear that there is a need to continue the development of green extraction systems coupled with on-line analysis systems for solid matrices of vegetable origin. Through the coupling of more advanced techniques, it is possible to obtain several benefits, including shorter operating time, ease of operation, better reproducibility, improved detection limits, greater sensitivity, and reliability of the results.

A common trend in current extraction and analysis technologies is the search for replacing the use of toxic reagents with green reagents obtained from renewable sources and the integration of extraction processes and analytical operations, saving energy and reducing the use of reagents. For chromatography, the main analysis technique that provides a lot of detailed information, alternative reusable solvents, such as eutectic solvents and ionic liquids, are sought for use as a mobile phase. The development of chromatography can reduce analysis time, improve separations, and obtain more accurate information. This evolution is carried out through the elaboration of more efficient columns, with smaller lengths and smaller filling particles. Additionally, the use of SFC to replace the LC mainly in normal phase and the enhancement of detectors are key points for on-line detection.

On-line detection can be used not only to monitor the process, but also to characterize samples and extracts in real time, providing greater quality and speed in obtaining information. Through the advancement of bioinformatics and artificial intelligence, a greater automation of processes will be possible, making it easier to calibrate equipment to identify certain failures, and allowing for the ability to interrupt or make adjustments to the process that is currently taking place.

In addition, the use of the coupling of these technologies can be a relevant strategy for the development of a circular economy. The benefits of the revalorization of agro-industrial waste are remarkable, and the establishment of biorefineries producing high value-added ingredients makes it possible to take full advantage of vegetable matrices.

## 6. Conclusions

Research on the automation and coupling of extraction processes with on-line detection started in 1990. The total number of publications reached the mark of 2353 articles, and since 1995, more than 60 publications are made annually, except in the last year of 2020. Spain published most of these studies, while the USA had the largest network of international partnerships. The most cited articles are primarily reviews, and their volume of information (among other aspects) is one of the reasons for the high number of citations. Advanced extraction techniques for solid matrices on-line systems coupled to chromatography (LC, GC, and SFC) are usually performed in dynamic mode with innovative techniques, such as SFE, UAE, MAE, PLE, and SubCWE. On-line coupling systems employ intermediate trapping, multiport valves, and one or more pumps for the dynamic extraction or transfer of the extract to the chromatographic system. The on-line coupling of an extraction to a chromatographic technique allows the entire analysis to be performed in a closed system. The main advantages are improved sensitivity, minimal sample contamination, and the possibility of a fully automated analytical system. For solid samples, more complex solutions are required for on-line analysis than for liquid samples. It usually requires some adaptation and optimization, as well as special knowledge of the underlying principles. However, the time invested in optimizing conditions is quickly paid back in terms of more efficient sample throughput, better reproducibility, and improved sensitivity.

## Figures and Tables

**Table 1:** Top 10 journals ranked by number of publications.

Rank	Journals	Country	Publishing Company	Impact Factor	Records	% of 2353
1	*Journal of Chromatography A*	Netherlands	Elsevier	4.759	374	15.895
2	*Talanta*	England	Elsevier	6.057	145	6.162
3	*Journal of Chromatography B*	Netherlands	Elsevier	3.205	144	6.120
4	*Analytica Chimica Acta*	Netherlands	Elsevier	6.558	133	5.652
5	*Analytical Chemistry*	USA	ACS Publications	6.986	88	3.740
6	*Analytical and Bioanalytical Chemistry*	Germany	Springer	4.157	77	3.272
6	*Chromatographia*	Germany	Springer	2.044	77	3.272
8	*Journal of Pharmaceutical and Biomedical Analysis*	Netherlands	Elsevier	3.935	72	3.060
9	*Electrophoresis*	Germany	WILEY	3.535	66	2.805
10	*Journal of Separation Science*	Germany	WILEY	3.645	61	2.592

The impact factor measures the average number of citations received in a particular year by papers published in the journal during the two preceding years. Journal impact factor (Clarivate Analytics, 2021) [36].

**Table 2:** Top 10 articles ranked by number of citations.

Rank	Title	Journals	Citations	Article Type	References
1	Phosphorus flame retardants: properties, production, environmental occurrence, toxicity and analysis	*Chemosphere*	1424	Review article	[37]
2	Solid-phase extraction: method development, sorbents, and coupling with liquid chromatography	*Journal of Chromatography A*	835	Review article	[38]
3	Green Analytical Chemistry	*Trends in Analytical Chemistry*	594	Review article	[39]
4	Metal-Organic Frameworks for Analytical Chemistry: From Sample Collection to Chromatographic Separation	*Accounts of Chemical Research*	548	Review article	[40]
5	Supercritical fluid extraction in herbal and natural product studies—a practical review	*Talanta*	434	Review article	[41]
6	Supercritical fluid extraction in plant essential and volatile oil analysis	*Journal of Chromatography A*	396	Review article	[42]
7	Recent advances in high-throughput quantitative bioanalysis by LC-MS/MS	*Journal of Pharmaceutical and Biomedical Analysis*	373	Review article	[43]
8	A general approach to desalting oligosaccharides released from glycoproteins	*Glycoconjugate Journal*	365	Research article	[44]
9	Automated online column-switching HPLC-MS/MS method with peak focusing for the determination of nine environmental phenols in urine	*Analytical Chemistry*	324	Research article	[45]
10	Serum concentrations of 11 polyfluoroalkyl compounds in the US population: Data from the National Health and Nutrition Examination Survey (NHANES) 1999–2000	*Environmental Science & Technology*	317	Research article	[46]

**Table 3:** Top 30 most frequent keywords (author keywords and keywords plus).

Rank	Author Keywords	Occurrences	Rank	Keywords Plus	Occurrences
1	*Solid-phase extraction*	266	1	*Solid-phase extraction*	671
2	On-line solid-phase extraction	159	2	*High performance liquid-chromatography*	414
3	*Mass spectrometry*	117	3	*Liquid-chromatography*	286
4	*Capillary electrophoresis*	102	4	*Mass-spectrometry*	275
5	*Pesticides*	96	5	*Tandem mass-spectrometry*	214
6	*Liquid chromatography*	87	6	Samples	213
7	On-line SPE	82	7	*Gas-Chromatography*	209
8	LC-MS/MS	77	8	Separation	193
8	*HPLC*	77	9	*Water*	172
8	*Supercritical fluid extraction*	77	10	Metabolites	144
8	Water analysis	77	11	Chromatography	135
12	*Sample preparation*	74	12	*Human plasma*	134
13	Column Switching	63	13	*Urine*	114
14	*Urine*	62	14	*Preconcentration*	112
15	*High performance liquid chromatography*	57	15	Microextraction	111
16	Environmental analysis	54	16	*Pesticides*	106
17	Automation	45	17	Polycyclic aromatic-hydrocarbons	103
17	Column liquid chromatography	45	18	*plasma*	102
19	On-line preconcentration	42	19	Identification	100
20	*Preconcentration*	40	20	*HPLC*	97
21	*Gas chromatography*	36	21	*Extraction*	88
22	*Water*	35	22	*Sample preparation*	85
23	LC-MS	32	23	*Capillary-electrophoresis*	83
24	On-line extraction	31	23	Quantification	83
25	*Water samples*	30	25	*Supercritical-fluid extraction*	82
26	*Extraction*	29	26	*Water samples*	74
26	*Plasma*	29	26	Waste-water	74
26	Monolithic column	29	28	On-line	72
29	*Human plasma*	28	28	Residues	72
29	*Tandem mass spectrometry*	28	30	drugs	70

*Italics* used for words present in the two columns of the table.

**Table 4:** Comprehensive report of SFE systems coupled with on-line analysis systems for solid matrices from vegetable origin.

Sample	Compounds of Interest	Coupled Techniques	Solvents	SFE Conditions (F; P; T)	SFE Method (Static Mode)	SFE Method (Dynamic Mode)	SFC/LC Method	Detector	References
Chilli Peppers	Carotenoids and apocarotenoids	SFE-SFC- QqQ/MS	CO_2_ (A) CH_3_OH (B)	2.0 mL/min; 150 bars; 80 °C	0–3 min, 10% B	3–4 min, 0% B	4–6 min, 0% B; 6–21 min, 0–80% B; 21–22 min, 80–100% B; 22–24, 100% B	APCI-MS	[54]
Green, yellow, and red bell peppers	Capsaicin	SFE-SPE-LC-MS	CO_2_ (A) CH_3_OH (B)	5.0 mL/min; 15 MPa; 50 °C	0–4 min, 5% B	4–8 min, 5% B	0 min, 45% B; 0–10 min, 80% B; 10–12 min, 100% B; 12–13.5 min, 100% B; 15.5–13.6 min, 45% B	ESI-MS	[55]
Yellow tamarillo fruits	Apocarotenoids and carotenoids	SFE-SFC- QqQ/MS	CO_2_ (A) CH_3_OH (B)	2.0 mL/min; 150 bars; 80 °C	0–3 min, 5% B	3–4 min, 10% B	4–6 min, 0% B; 6–14 min, 0–40% B; 14–16 min, 40% B	APCI-MS	[56]
*Chaenomelis Fructus*	Oleanoic acid and ursolic acid	SFE-SFC	CO_2_ (A) CH_3_OH (B)	5.0 mL/min; 15 MPa; 35 °C	0–1 min, 20% B	1–8 min, 5% B	0–10 min, 5–10% B; 10–14 min, 10% B; 14–16 min, 10–40% B; 16–20 min, 40% B	PDA	[16]
Microalgae	Carotenoids, chlorophyll A, ergosterol, and total lipids	SFE-UV/Vis- ELSD	CO_2_ (A) CH_3_CH_2_OH (B)	1.5 mL/min; 15–30 MPa; 40–60 °C	-	-	-	UV/Vis ELSD	[57]
Vanilla beans	Aromatic constituents	SFE-SFC	CO_2_ (A) CH_3_OH (B)	2.0 mL/min 10–20 MPa; 35–55 °C	-	-	0–13 min, 2–10% B; 13–17 min, 10–15% B; 17–18 min, 15% B	PDA	[58]
Red Habanero peppers	Carotenoids	SFE-SFC- QqQ/MS	CO_2_ (A) CH_3_OH (B)	3.0 mL/min; 150 bars; 40–80 °C	0–3 min, 10% B	3–4 min, 0% B	4–6 min, 0% B; 6–14 min, 0–40% B; 14–16 min, 40% B	APCI-MS	[59]
Linseed	Lipids	SFE–ELSD	CO_2_ (A) CH_3_CH_2_OH (B)	1.5 mL/min 30 MPa; 80 °C	-	-	-	ELSD	[60]

**Table 5:** Comprehensive report of UAE systems coupled with on-line analysis systems for solid matrices from vegetable origin.

Sample	Compounds of Interest	Coupled Techniques	Solvents for UAE	Mobile Phase for LC	UAE Conditions (F; P; T)	Reference
*Porous fungus, P. vaninii*	Phytochemicals	UAE and on-line extraction.	n-Hexane–ethyl acetate–acetonitrile–water (5.5:2.5:5.0:0.4, *v*/*v*/*v*) was used as the solvent system	elution procedure: 0–20 min, 45%–80% (acetonitrile), 65%–10% (water); flow rate: 0.4 mL/min	500 W, 170 mL/min 40 °C	[77]
*Frangula alnus* (cortex) and *Rubia tinctorum* (roots and rhizomes)	Anthraquinones	UAE + spectrophotometric	Triton X-100	Non-applicable	325 W, 35 kHz at 75 °C for 10 min	[78]
*Rhodiola rosea*	*Rhodiosin*	UAE+SPE coupled UPLC	Ethanol:water (95%)	ACN-formic acid (0.1%, *v*/*v*): 0–8 min, 5–40%, acetonitrile; 8–15 min, 40–100% acetonitrile. The flow rate of 0.3 mL/min	60 kHz, 360 W on the scale of 0–100	[79]
*Scutellaria baicalensis* Georgi	Flavonoids	UAE-HPLC	Ethanol:water (60%)	ACN-water with 0.1% phosphoric acid. The gradient conditions was 0–15 min, 20–30% ACN; 16–20 min, 30–50% ACN; 21–28 min, 50–20% CAN. The flow rate of 1 mL/min	40 kHz, 150 W	[72]
basil (*Ocimum basilicum L.*), oregano (*Origanum vulgare L.*), rosemary (*Rosmarinus officinalis L.*), sage (*Salvia officinalis L.*), spearmint (*Mentha spicata L*.) and thyme (T*hymus vulgaris*).	Phenolic acids	UAE+SPE coupled on HPLC	Ethanol:water (60%)	0 min 5% methanol, 2 min 5% methanol, 6 min 25% methanol, 13 min 40% methanol, 26 min 40% methanol. The flow rate of 1 mL/min	Flow rate 0.25 mL/min, temperature 45 °C and extraction time 15 min.	[73]
Textile fragments	Formaldehyde	UAE coupled on -HPLC	Water	4 mmol L^−1^ sodium dihydrogen phosphate in 50% ACN at a flow rate of 1.0 mL min^−1^.	40 kHz. 100 and 800 W, 80 °C.	[74]
Colistin in feed.	colistin A and B	UAE coupled with HPLC	-	-	-	[80]

**Table 6:** Comprehensive report of MAE systems coupled with on-line analysis systems for solid matrices from vegetable origin.

Sample	Compounds of Interest	Coupled Techniques	Solvents	MAE Conditions (F; P; T)	LC/GC Method	Detector	Reference
*Salvia miltiorrhiza* Bunge	Lipophilic constituents (tanshin-one I, cryptotanshinone, and tanshinone IIA)	DMAE-HPLC	[C6MIM]Cl aqueous solution (A), Ethanol (B)	1.6 mL/min; 180 W	methanol-water (*v*/*v*, 81/19) and isocratic elution; 0.5 mL min^−1^; at 40 °C and injection volume of 20 μL.	Photodiode-array detector (PAD)	[85]
Mushroom	Nicotine	HTDMAE- SPE	Water, elution solvent (methanol–ammonia, 95:5, *v*/*v*)	2.0 mL/min; 1000 W	20 mmol L^−1^ ammonium acetate solution (pH = 3) and methanol (80:20, *v*/*v*)); 1.0 mL min^−1^, at 30 °C and injection volume of 20 μL.	UV detector monitoring at 260 nm	[49]
Tea	Caffeine	DMAE coupled on-line with clean-up	Ethanol	1.0 mL/min; 70 W	30% methanol and 70% water; 1.0 mL min^−1^.	UV detector monitoring at 270 nm	[86]
Andrographis paniculata Nees	Andrographolide and dehydroandrographolide	DMAE-HPLC	Methanol	1.0 mL/min; 80 W	65% aqueous methanol; 1.0 mL min^−1^ and injection volume of 20 μL.	Photodiode-array detector (PAD)	[87]
Grain samples, including wheat, rice, corn and bean	Organochlorine pesticides	DMAE–SPE–HPLC	95% acetonitrile	1.0 mL/min; 80 W	Mobile phase of 75% ACN aqueous solution; 1.0 mL min^−1^.	---	[51]
Flos Carthami	Safflower yellow	MAE-UV	60% methanol	1.0 mL/min; 80 W	---	---	[88]

**Table 7:** Comprehensive report of PLE systems coupled with on-line analysis systems for solid matrices from vegetable origin.

Sample	Compounds of Interest	Coupled Techniques	PLE Conditions (F; P; T)	Solvents	LC Method	Detector	Reference
Yerba mate (*Ilex paraguariensis*)	Alkaloids, phenolic acids and flavonoids	PLE-SPE- HPLC	2.0 mL/min; 100 bars; 40–80 °C	H_2_O (A) CH_3_CN (B)	1 min, 10% B; 2 min, 20% B; 4 min, 30% B; 5 min, 90% B; 8 min, 10% B	UV-Vis	[92]
Strawberry and apple	Herbicide 2-(3-chlorophenoxy) propionic acid (3-CPA)	µPLE-SPE- HPLC	1.0 mL/min; 30 bars; 150–160 °C	H_2_O (A) CH_3_CN (B)	70% A/30% B/0.1% Formic Acid	UV-Vis	[93]
Black tea	Gallic ccid, caffeine, and flavonols	PLE-SPE- HPLC	2.0 mL/min; 100 bars; 40–80 °C	H_2_O (A) CH_3_CN (B)	0 min, (95% A); 1 min, (95% A); 3 min, (90% A); 7 min, (87.5% A); 9 min, (85% A); 10 min, (82% A); 18 min, (77% A); 20 min, (0% A); 22 min, (0% A); 23 min, (95% A)	UV-Vis	[94]
Dried root (*Polygonum viviparum*)	Antioxidants	PLE-HPLC	1.0 mL/min; 70 °C	H_2_O (A) CH_3_CN (B)	0–5 min, 0% B; 5–6 min, 5% B; 6–21 min, 15% B; 21–30 min, 20% B; 30–35 min, 80% B; 35–37 min, 0% B	PDA	[95]
Ginseng of the desert (*Cistanche deserticola*)	Primary phenylethanoid glycosides	PLE–TFC–HPLC	2.5 mL/min; 13 MPa; 70 °C	H_2_O (A) CH_3_CN (B)	0–3 min, 10% B; 3–10 min, 10–20% B; 10–25 min, 20–30% B; 25–35 min, 30–45% B; 35–40 min, 45–60% B; 40–45 min, 60–90% B; 45–48 min, 90% B; 48, 1–60 min, 10% B	PDA	[96]

## Data Availability

Not applicable.

## Supplementary Materials

The following supporting information can be downloaded at: https://www.mdpi.com/article/10.3390/molecules27196272/s1, Figure S1: Methodological synthesis applied to the bibliometric analysis.
