# Supplementary material for: Green Extraction Processes for Complex Samples from Vegetable Matrices Coupled with On-Line Detection System: A Critical Review
Source: Molecules. 2022 Sep 23;27(19):6272. doi: 10.3390/molecules27196272 (PMC9571248; doi:10.3390/molecules27196272)
Supplement: Supplementary file 1 [file molecules-27-06272-s001.zip › molecules-1903554-supplementary.pdf]

SUPLEMENTARY MATERIAL

Green Extraction Processes for Complex Samples from Vegetable Origin Coupled with On-line Detection System: A Critical review

Francisco W. Maciel-Silva[1], Daniel Lachos-Perez[2], Luz Selene Buller[1], William Gustavo Sganzerla[1], Montserrat Pérez[3], Mauricio A. Rostagno[4*], Tânia Forster-Carneiro[1]

[1]*School of Food Engineering (FEA), University of Campinas (UNICAMP), Rua Monteiro Lobato, n.80, 13083-862 Campinas, São Paulo, Brazil.*

[2]*Department of Chemical Engineering, Federal University of Santa Maria, 1000, Roraima Avenue, Santa Maria, RS, 97105-900, Brazil.*

[3]*Department of Environmental Technologies, University of Cadiz, Campus de Puerto Real, s/n. 11500 Puerto Real, Cadiz, Spain*

[4]*School of Applied Sciences (FCA), University of Campinas (UNICAMP), Rua Pedro Zaccaria, n. 1300, 13484-350, Limeira, Sã o Paulo, Brazl.*

* Corresponding author:

E-mail address: mauricio.rostagno@fca.unicamp.br

WEB OF SCIENCE

⇩

Search mode: Topic
Refining: Articles and Reviews
Period: 1990 – 2021
Search terms:
((“Solid Phase Extraction” OR “Liquid-liquid Extraction” OR “Supercritical Fluid Extraction” OR “Pressurized Liquid Extraction” OR “Supercritical Water” OR “Subcritical Water” OR “Accelerated Solvent Extraction” OR “Microwave Assisted Extraction” OR “Ultrasound Assisted Extraction”)
AND
(“Chromatography” OR “HPLC” OR “LC” OR “GC” OR “UV-detection” OR “Supercritical Fluid Chromatography”)
AND
(“On-line” OR “In-line”)) **2,353 Archives**

⇩

BIBLIOMETRIC ANALYSIS

⇩

Publications: Annual and total number of publications
Scientific activity: Top 6 research areas
International collaboration: Top 30 countries
Journals: Top 10 journals
Citation: Top 10 most cited articles
Relevance to search: Top 30 author’s keywords and keywords plus

Figure S1. Methodological synthesis applied to the bibliometric analysis.
